# Pyogenic liver abscess in the North Denmark Region - a population-based cohort study (2010–2022)

**DOI:** 10.1007/s10096-025-05307-1

**Published:** 2025-10-17

**Authors:** Margarita Dudina, Søren Schou Olesen, Kirstine K. Søgaard, Hans Linde Nielsen

**Affiliations:** 1 Department of Clinical Microbiology, Aalborg University Hospital, Hobrovej 18-22, Aalborg, 9000 Denmark; 2 Department of Clinical Microbiology, Aarhus University Hospital, Aarhus N, 8200 Denmark; 3 Department of Clinical Medicine, Aalborg University, Gistrup, 9260 Denmark; 4 Centre for Pancreatic Diseases, Department of Gastroenterology and Hepatology, Aalborg University Hospital, Aalborg, 9000 Denmark

**Keywords:** Pyogenic liver abscess, Liver infection, Infected liver cyst, Epidemiology, Population-based

## Abstract

**Purpose:**

To examine the incidence, clinical characteristics, and outcomes of pyogenic liver abscess (PLA) in a population-based cohort.

**Methods:**

We conducted a population-based cohort study of patients diagnosed with PLA in the North Denmark Region from 2010 to 2022. Cases were identified using ICD-10 discharge code (K75.0) and/or microbiology samples, followed by medical record review. We estimated incidence rates, 30-day mortality, and used Cox regression to estimate hazard ratios (HRs) for all-cause mortality within 365 days, stratified by clinical and microbiological factors.

**Results:**

We identified 249 patients (56% male) with a median age of 68 years (IQR, 59–77). The mean annual incidence was 3.1 per 100,000 person-years, increasing from 2.9 in 2010 to 4.8 in 2022. The most common symptoms were fever (57%) and abdominal pain (48%). Biliary tract disease was the most frequent source, accounting for 35%, while 31% were cryptogenic. A microbiological diagnosis was established in 171 patients (69%), most often isolating *Escherichia coli* and *Streptococcus anginosus* group. Drainage was performed in 73% of cases, and piperacillin/tazobactam was the most used intravenous antibiotic. The 30-day mortality was 5%, rising to 22% at one year. In multivariable analysis, malignancy (HR 3.19, 95% CI: 1.30–7.82) and polymicrobial abscess cultures (HR 4.15, 95% CI: 1.23–14.07) were associated with increased 365-day all-cause mortality. In contrast, drainage, infected cysts, multifocal abscesses, and blood culture positivity were not.

**Conclusion:**

PLA incidence increased over the study period. While short-term mortality was low, one-year mortality was high among patients with malignancy or polymicrobial infection.

**Supplementary Information:**

The online version contains supplementary material available at 10.1007/s10096-025-05307-1.

## Introduction

Pyogenic liver abscess (PLA) is a serious and potentially life-threatening infection characterised by a localised collection of pus within the liver parenchyma caused by bacterial pathogens [1]. Clinically, it presents with a broad spectrum of manifestations, ranging from nonspecific symptoms such as fever, chills, abdominal pain, and weight loss to more severe signs of systemic sepsis [2, 3].

Historically, PLA was associated with high mortality rates, often exceeding 50% [4], and despite improvements in imaging techniques and medical care, PLA continues to pose a clinical challenge. The presentation is heterogeneous [5], and a multidisciplinary approach involving a hepatologist, radiologist, and clinical microbiologist is often essential for identifying the source of infection and establishing an effective treatment plan [1]. However, diagnostic difficulties persist, particularly in differentiating PLA from other hepatic pathologies, such as cysts or necrotic metastases, which may present with overlapping imaging features [1].

The incidence of PLA varies considerably by geography. Higher rates have been reported in Asia, with South Korea and Taiwan reporting incidences of 14.4 and 17.6 cases per 100,000 person-years, respectively [6, 7]. In contrast, lower incidences are observed in Western countries, including 4.1 per 100,000 person-years in the United States, 5.0 in New Zealand, 3.4 in Sweden, and 7.0 in Germany [5, 8–10]. In Denmark, a nationwide study from 1977 to 2002 reported an increasing incidence, rising from 0.6 to 1.8 cases per 100,000 person-years in men and from 0.8 to 1.2 in women [2], but recent data are not available.

We recently validated the Danish ICD-10 diagnosis code K75.0 for PLA in the North Denmark Region, confirming a positive predictive value (PPV) of 77% (95% CI: 72–82%) [11]. In the present study, using this validated cohort supplemented by microbiological data, we aimed to describe the incidence, clinical presentation, microbiological and imaging findings, treatment modalities, and all-cause mortality associated with PLA over a 12-year period.

## Materials and methods

### Study design and setting

We conducted a population-based cohort study of patients diagnosed with PLA in the North Denmark Region between January 1, 2010, and June 30, 2022. The North Denmark Region comprises a stable population of approximately 590,000 inhabitants, representing approximately 10% of the Danish population [12]. All residents have equal access to tax-financed health services, including hospital care. Each Danish citizen is assigned a unique 10-digit civil registration number (CPR), which enables unambiguous linkage across medical and administrative registries, allowing comprehensive follow-up [13]. All included patients were treated at Aalborg University Hospital or the North Denmark Regional Hospital, including smaller satellite hospitals [11].

## Data sources

We utilised data from the Business Intelligence and Analysis Unit of the North Denmark Region to identify all patients with a discharge diagnosis of PLA, as defined by the 10th revision of the International Statistical Classification of Diseases and Related Health Problems (ICD-10) code K75.0 (Abscess of liver), during the study period. In addition to registry data, we accessed the regional microbiological database (WWBakt, Autonik AB, Nyköping, Sweden), maintained by the Department of Clinical Microbiology, Aalborg University Hospital, which provides diagnostic microbiology services for the entire region. From this database, we retrieved results of positive microbiological specimens from abscess material and blood cultures to identify additional cases of PLA not captured through ICD-10 coding alone.

We conducted a comprehensive review of patients’ medical records to confirm diagnoses and extract detailed information, including clinical presentation, comorbidities, risk factors, laboratory and imaging findings, antimicrobial therapy, and follow-up data. Follow-up included the date of death and/or rehospitalisation for up to 365 days following hospital admission, with 30 June 2023 as the end of the study follow-up period. Recent travel exposure, defined as travel outside Denmark, within 30 days prior to hospital admission, was also recorded, regardless of travel purpose. All data was registered in an electronic case report form using the Research Electronic Data Capture (REDCap) system [14].

## Case definition and diagnostic criteria for PLA

The diagnosis of PLA was confirmed if any of the following criteria were met [11]: (i) Imaging evidence of a liver abscess combined with culture-positive abscess material and/or PCR detection of pathogenic organisms. (ii) Imaging evidence of a liver abscess and a positive blood culture, in the absence of microbiological confirmation from abscess material. (iii) Imaging evidence of a liver abscess and a clear clinical and/or radiological response to antimicrobial therapy despite negative cultures. (iv) Histopathological confirmation of PLA, regardless of culture or imaging findings.

## Microbiological samples

The microbiological methods have been described in detail elsewhere [11]. In brief, pus samples were cultured on standard agar media and incubated under both aerobic and anaerobic conditions. Blood cultures were processed using the Bact/ALERT system (BioMérieux, Marcy-l’Étoile, France) from 2010 to 2015 and subsequently with the BACTEC FX Top system (Becton Dickinson, Franklin Lakes, NJ, USA). Bacterial identification was performed using conventional biochemical tests and MALDI-TOF mass spectrometry (Bruker, Bremen, Germany). Antimicrobial susceptibility testing was carried out according to the European Committee on Antimicrobial Susceptibility Testing (EUCAST) guidelines. In selected cases, 16S/18S rRNA gene sequencing was performed on pus samples at the national reference laboratory, Statens Serum Institut, Denmark [15].

### Statistical analysis

Continuous variables are presented as medians with interquartile ranges (IQRs), and categorical variables are presented as frequencies with percentages. Annual incidence rates were calculated as the number of incident cases per 100,000 person-years, based on yearly population data from the North Denmark Region [12]. To examine the association between clinical and microbiological factors and all-cause mortality within 365 days of PLA diagnosis, we performed univariable and multivariable Cox proportional hazards regression analyses. The multivariable model was adjusted for age, sex, and comorbidities (diabetes and cardiovascular disease). Results are reported as Hazard ratios (HRs) with 95% confidence intervals (CIs). In stratified analysis, we investigated the impact of abscess type (PLA not associated with malignancy or cyst (reference group), infected tumour/metastasis, or infected liver cyst), abscess focality (unifocal vs. multifocal), blood or abscess culture results (negative, monomicrobial, or polymicrobial), and abscess drainage (yes, no) on mortality. Kaplan–Meier survival curves were generated to illustrate cumulative all-cause mortality according to abscess type and culture results. All analyses were conducted using Stata/MP version 18.0 (StataCorp, College Station, TX, USA).

### Ethics

The study was approved by the Regional Council of the North Denmark Region (approval no. 2022–025705) and registered in the region’s research registry (ID: F2022-138) in accordance with Danish Data Protection Agency regulations.

## Results

A total of 297 patients were assigned the diagnosis code K75.0 during the study period. Five patients were excluded due to initial hospitalisation outside the North Denmark Region, and 67 were misclassified, as previously reported [11]. In parallel, the regional microbiology database identified 448 specimens from 283 patients, categorised as liver pus. Among these, 120 overlapped with patients identified via the ICD-10 code, while 139 patients did not have a PLA diagnosis code assigned. Following a detailed review of the medical records and application of the predefined diagnostic criteria, an additional 24 patients were confirmed as PLA cases. Consequently, the final study cohort comprised 249 patients with PLA during the study period. According to the diagnostic criteria: (i) 140 patients had both imaging and microbiological confirmation, (ii) 31 patients had imaging confirmation with a positive blood culture, (iii) 72 patients had imaging confirmation with a clinical response to antimicrobial therapy, and (iv) six PLA cases were confirmed by histopathology. The inclusion and exclusion process is summarised in Supplementary Fig. 1.

## Incidence

**Fig. 1:**
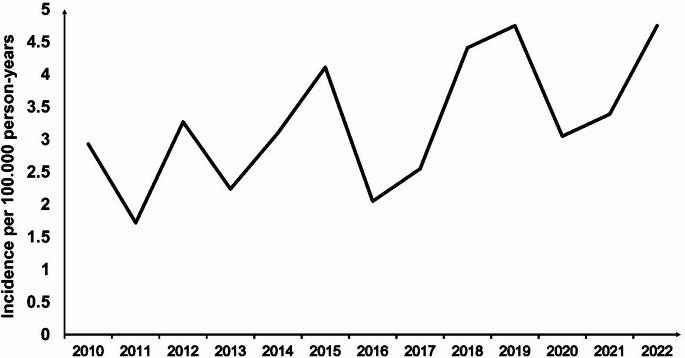
Annual incidence of PLA in the North Denmark Region from 1 January 2010 to 30 June 2022

The mean annual incidence of PLA during the study period was 3.1 per 100,000 person-years, with an upward trend over time, increasing from 2.9 per 100,000 person-years in 2010 to 4.8 per 100,000 person-years in the first half of 2022 (Fig. 1).

### Patient characteristics

The median age of the PLA cohort was 68 years (IQR: 59–77), and 138 (56%) were men (Table 1). The age distribution is shown in Supplementary Fig. 2. The most common comorbidities were cardiopulmonary disease (33%), malignancy (33%), and diabetes mellitus (19%). Among patients with malignancy, the majority had a recent history of colorectal cancer (35%), followed by pancreatic cancer (22%). Notably, of those with colorectal cancer, 23 (79%) had liver metastases, and 14 (61%) of these developed PLA. Finally, 17% of patients were described as previously healthy, with no chronic conditions recorded.

**Table 1:** Patient characteristics of patients (*n* = 249) with PLA in the North Denmark Region, between January 1, 2010, and June 30, 2022

Variable	Value
Demographics	
Age, total (median [IQR])	68 [59–77]
Age by sex, (median [IQR])	Male: 68 [56–75] Female: 69 [59–81]
Sex distribution, n (%)	Male: 138 (56%) Female: 111 (44%)
Underlying diseases, n (%)	
Cardiopulmonary disease	83 (33)
Diabetes mellitus^a^	46 (19)
Malignancy	81 (33)
Colorectal	28 (35)
Pancreas	18 (22)
Cholangiocarcinoma	10 (12)
Lung cancer	5 (6)
Breast cancer	4 (5)
Skin cancer	4 (5)
Hepatocellular carcinoma	3 (4)
Hematological malignancy	3 (4)
Other^b^	54 (22)
Clinical presentation n (%)	
Fever (≥ 38 °C)	141 (57)
Abdominal pain	119 (48)
Nausea	79 (32)
Chills	49 (20)
Malaise	47 (19)
Weight loss	33 (13)
Jaundice	3 (1)
Duration of symptoms before admission, (Median days [range])	5 [2–14]
Travel history^c^ (within 30 days), n (%)	7 (3)
Length of hospitalisation, (median days [IQR])	17 [10–27]

^a^ Diabetes mellitus type I (*n* = 8), Diabetes mellitus type II (*n* = 38)

^b^ Including diverticulitis (*n* = 5), rheumatoid arthritis (*n* = 5), cirrhosis (*n* = 3), and inflammatory bowel disease (Crohn’s disease, *n* = 2; ulcerative colitis, *n* = 1)

^c^ Travel history included (Thailand (*n* = 2), Turkey (*n* = 2), Greece (*n* = 1), Philippines (*n* = 1), United States (*n* = 1)

The median duration of symptoms prior to hospital admission was 5 days (IQR: 2–14), with a maximum of 180 days. The most common presenting symptoms were fever and abdominal pain, reported in 57% and 48% of the patients, respectively (Table 1). Jaundice was rare, documented in only three patients (1.2%). Notably, 12% of patients were admitted without either fever or abdominal pain; among these, seven (24%) were diagnosed incidentally during planned abdominal surgery.

Biochemical parameters are presented in Supplementary Table 1. Abnormal findings included elevated C-reactive protein in 97% of patients, increased white blood cell count in 78%, elevated alkaline phosphatase in 78%, and decreased albumin levels in 84%.

### Imaging and anatomical location of PLA

A total of 239 (96%) patients underwent evaluation with computed tomography (CT). Supplementary imaging included magnetic resonance imaging (MRI) in 32 (13%) patients and positron emission tomography–CT (PET-CT) in 16 (6%). Abdominal ultrasound was performed in 212 (85%) patients, with 110 (52%) of these conducted after CT imaging, primarily to guide drainage procedures. In cases where imaging was inconclusive for PLA or used to exclude underlying malignancy, a diagnostic liver biopsy was carried out in eight (3%) patients.

The majority of patients (*n* = 151, 60%) had a single abscess, most commonly located in the right hepatic lobe (*n* = 105, 70%). The mean diameter of a solitary hepatic abscess was 5.8 cm (range: 1–13 cm). Two abscesses were observed in 24 (10%), and three abscesses in 11 (4%) patients. Multiple lesions (more than three) were identified in 63 (25%) patients.

### Risk factors for PLA

Gallbladder and/or bile duct diseases, including cholelithiasis, choledocholithiasis, cholecystitis, cholangitis, cholangiocarcinoma, or recent biliary tract surgery, were identified as the source of infection in 35% of patients, as documented in the medical records by the treating physicians during routine clinical care (Table 2). A portal origin of infection was identified in 20% of patients, including cases associated with intra-abdominal abscesses, pancreatitis, or diverticulitis. Infection occurred in 8% of patients with primary liver tumours or liver metastases, and likewise in 8% of patients with bacterial superinfection of liver cysts. In the remaining 31% of patients, the cause of PLA was not identified and was therefore classified as cryptogenic.

**Table 2:** Risk factors associated with the development of pyogenic liver abscess

Condition	*n* (%)
Cryptogenic	77 (31)
Biliary	88 (35)
Gallstone diseases	45 (49)
Recent history of biliary tract surgery	26 (28)
Cholangitis	32 (35)
Cholangiocarcinoma	12 (13)
Portal vein seeding	50 (20)
Diverticulitis	21 (8)
Intraabominal abscesses	18 (7)
Pancreatitis	15 (6)
Superinfection in primary liver tumor/metastasis	21 (8)
Infected liver cyst	20 (8)
Infection after operation in liver parenchyma	8 (3)
Hematogenic	4 (2)
Other (trauma, dental abscess)	3 (1)

Note: Patients could have more than one identified risk factor. Subcategories are shown as percentages of the primary group

### Microbiological identifications

A microbiological diagnosis was confirmed in 171 (69%) patients based on results from blood cultures or abscess material. Blood cultures were obtained from 236 (95%) patients, of which 101 (43%) yielded positive results. Among these, 68 (67%) were monomicrobial, and 33 (33%) were polymicrobial, i.e., the cultivation of two or more species. Of the 135 patients with negative blood cultures, 40 (30%) had the blood cultures collected after the initiation of antimicrobial therapy. Abscess material was collected from 180 (72%) patients, with positive cultures obtained in 140 (78%) cases. Monomicrobial growth was observed in 65 (46%), while polymicrobial growth was observed in 75 (54%). Seventy patients had both a positive blood culture and a positive abscess culture, and in 48 (69%) of these, the same pathogen was isolated from both sources.

The most frequently isolated pathogens in both blood and abscess cultures were *Escherichia coli* and the *Streptococcus anginosus* group. In blood cultures, *E. coli* and the *S. anginosus* group were identified in 33 (31%) and 18 (17%) patients, respectively. In abscess cultures, they were found in 48 (34%) and 30 (21%) patients, respectively. *Klebsiella pneumoniae* was identified in a total of 26 (10%) patients, whereas other commonly cultured microorganisms included *Enterococcus faecium*, *Bacteroides fragilis*, *Clostridium perfringens*, other obligate anaerobes, and *Candida* species (Supplementary Table 2).

Microbiome sequencing (16S/18S) of abscess aspirate was performed in 26 (11%) patients, with positive findings in 21 (81%), predominantly identifying obligate anaerobes. Sequencing contributed to the microbiological diagnosis in six patients with culture-negative PLA. Serological and molecular testing for *Entamoeba histolytica* and *Echinococcus* were performed in a small subset of patients, with all results yielding negative findings.

### Treatment

The median length of hospital stay was 17 days (IQR: 10–27). Drainage was performed in 183 (73%) patients, primarily via percutaneous drainage (*n* = 160, 87%) or needle aspiration (*n* = 23, 13%). Open surgery was required in four (2%) patients. Antimicrobial therapy was administered to 247 (99%) patients. One patient died before treatment initiation, and one received drainage alone. Intravenous (IV) antimicrobial therapy was administered to 236 (96%) patients, of whom 129 (52%) received a combination of IV and oral regimens. The median total duration of antimicrobial therapy was 23 days (IQR: 12–38; range: 1–88), comprising a median of 14 days (IQR: 7–21) of intravenous therapy and 9 days (IQR: 9–28) of oral treatment.

Piperacillin/tazobactam was the most used intravenous antibiotic, administered to 113 (46%) patients, either as monotherapy (*n* = 71, 63%) or in combination with metronidazole (*n* = 42, 37%). Cefuroxime plus metronidazole was used in 31 (13%) patients, and carbapenems were administered to 16 (7%) patients, of whom seven also received metronidazole. For oral therapy, ciprofloxacin was the most frequently prescribed agent, administered to 79 (32%) patients, including 37 (15%) who received it in combination with metronidazole. Amoxicillin with clavulanic acid was used in 26 (19%) patients.

### Outcomes and all-cause mortality

Six patients were lost to follow-up after discharge, as they resided outside the North Denmark Region. Among the remaining cohort, the majority (*n* = 177, 73%) were considered cured after the first episode of PLA, with no subsequent hospitalisations related to PLA during the one-year follow-up. However, 39 (16%) patients were readmitted with suspected PLA recurrence, of whom 30 (12%) had a confirmed recurrent PLA.

**Fig. 2:**
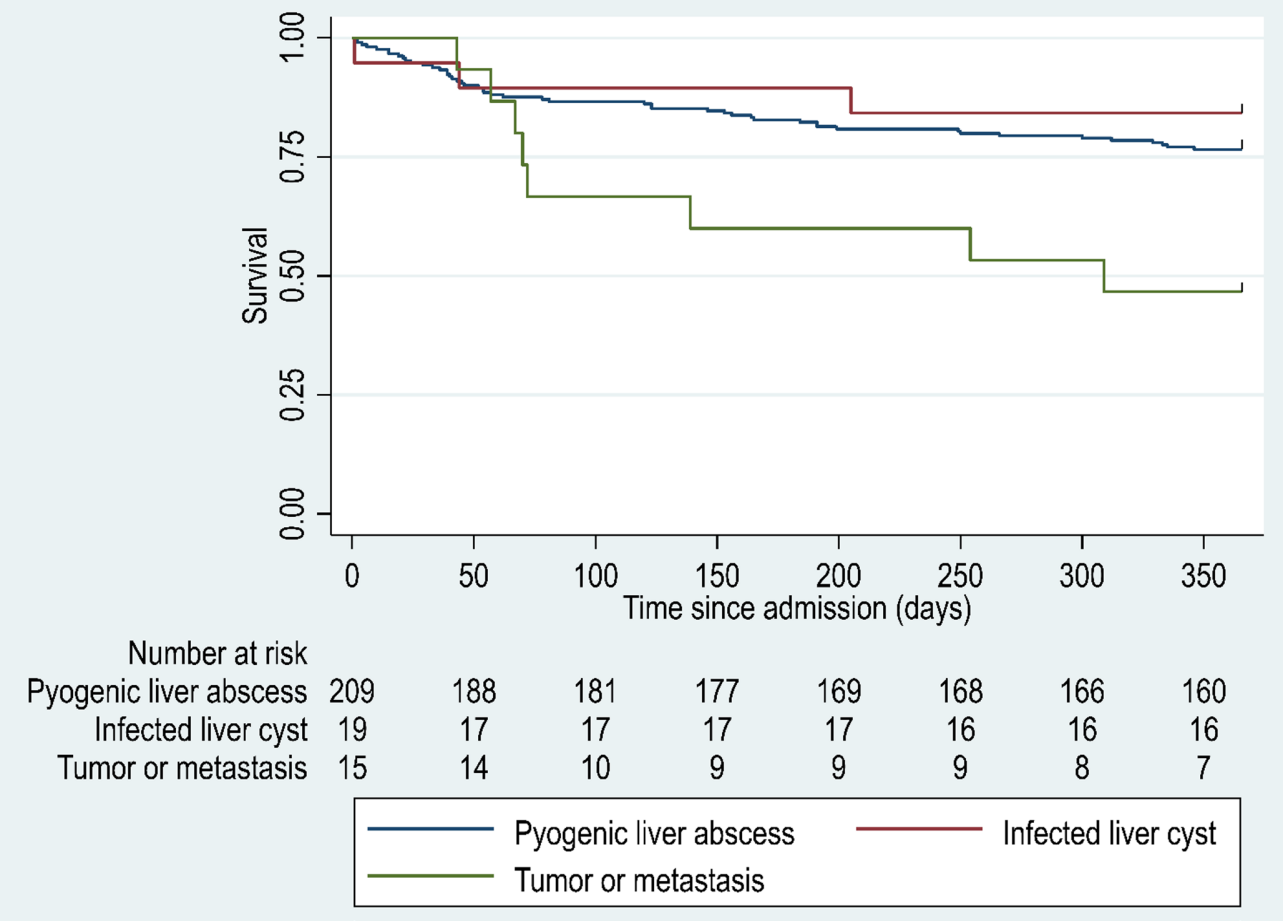
Kaplan–Meier survival curves for patients with PLA stratified by abscess type. The survival probability over 365-days following hospital admission is shown for patients with PLA not associated with malignancy or cyst (blue line), infected liver cysts (red line), and abscesses arising from tumours or metastases (green line). The number of patients at risk at each time point is indicated below the x-axis

In total, 60 (24%) patients (female/male: *n* = 25/35) died within 365 days of admission. Of these, 13 (5%) deaths occurred within the first 30 days. The 365-day all-cause mortality by abscess type is illustrated in Fig. 2. Patients with an infected tumour or metastasis had an increased risk of death compared to those with PLA not associated with malignancy (HR 3.19, 95% CI: 1.30–7.82) (Table 3). Similarly, polymicrobial growth from abscess cultures was associated with increased mortality compared to culture-negative abscesses (HR 4.15, 95% CI: 1.23–14.07) (Supplementary Fig. 3). Other variables, including infected liver cysts, multifocal abscesses, and blood culture results, were not significantly associated with 365-day mortality in either univariate or multivariable analyses (Table 3). There was no statistically significant association between abscess drainage and 365-day mortality (HR 0.71, 95% CI: 0.43–1.19).

**Table 3:** Cox proportional hazards regression analysis of factors associated with 365-day all-cause mortality. Hazard ratios (HRs) with 95% confidence intervals (CIs) were estimated using univariate and two^a, b^ multivariable Cox regression models. The reference group for abscess type was PLA not associated with malignancy or cyst. Abscess culture categories were based on Microbiological growth: negative, monomicrobial, or polymicrobial

	Univariate	Multivariate^a^	Multivariate^b^
	HR (95% CI)	HR (95% CI)	
Abscess type			
- Pyogenic liver abscess	1 (reference)	1 (reference)	1 (reference)
- Infected liver cyst	0.65 (0.20–2.10)	1.34 (0.39–4.56)	1.04 (0.30–0.54)
- Infected tumour or metastasis	2.58 (1.22–5.46)	3.50 (1.42–8.62)	3.19 (1.30–7.82)
Multifocal abscess (vs. unifocal)	1.07 (0.64–1.79)		
Blood culture^c^			
- Negative	1 (reference)		
- Monomicrobial	0.80 (0.41–1.57)		
- Polymicrobial	1.87 (0.96–3.67)		
Abscess culture^d^			
- Negative	1 (reference)	1 (reference)	1 (reference)
- Monomicrobial	2.31 (0.65–8.30)	1.94 (0.53–7.01)	2.28 (0.63–8.29)
- Polymicrobial	4.83 (1.46–16.01)	4.49 (1.32–15.26)	4.15 (1.23–14.07)
Abscess drainage (vs. no drainage)^e^	0.71 (0.43–1.19)		

^a^ Adjusted for age and sex

^b^ Adjusted for age, sex, diabetes and cardiovascular disease

^c^ Blood culture was not collected for 13 patients

^d^ Material for abscess culture was not collected for 69 patients

^e^ Abscess drainage refers to any form of drainage versus no drainage

## Discussion

This population-based study presents the epidemiology, clinical characteristics, imaging findings, microbiology, treatment modalities, and outcomes of patients with PLA in the North Denmark Region over a 12-year period. The mean annual incidence was 3.1 per 100,000 person-years, with an upward trend during the study period. Gallbladder or biliary tract disease was the most frequently identified anatomical source of infection, but in nearly one-third of cases, no apparent underlying cause could be established. The overall 30-day mortality was 5%, and 22% died within one year. Polymicrobial abscess cultures and underlying malignancy were associated with increased one-year mortality.

While the incidence varied over the study period, a general increasing trend was observed, reaching 4.7 cases per 100,000 person-years in the first half of 2022. This finding is consistent with those from a previous Danish study [2]. Similar upward trends have also been observed in recent studies from Southern Sweden, where the incidence nearly tripled from 1.8 per 100,000 person-years in 2011 to 5.2 per 100,000 person-years in 2020, and from South Korea, where rates rose from 5.7 to 14.4 per 100,000 between 2007 and 2017 [7, 9]. Several factors may explain this trend, including enhanced diagnostic accuracy, increased clinical awareness, rising prevalence of risk factors (e.g. malignancy and diabetes), and modifications in hospital coding practices. In contrast, data from Germany showed stable incidence rates, with approximately 7 cases per 100,000 person-years reported annually during 2013–2019 [10], suggesting potential regional differences in epidemiology, healthcare practices, or data capture methods.

The median age in our cohort was 68 years, with a slight male predominance, consistent with previous European studies [9, 10, 16]. Compared to the historical Danish study conducted from 1977 to 2002 [2], the median age of female patients has remained stable (68 vs. 69 years), while the median age of male patients has increased from 61 to 68 years.

Approximately one-third of patients had malignancies, primarily colorectal and pancreatic cancer. Among colorectal cancer patients, most had liver metastasis, of which over half developed secondary PLA. This supports previous studies showing an association between colorectal cancer, hepatic metastasis and PLA [17, 18]. Diabetes mellitus, a known risk factor for PLA and for poor prognosis, was present in 19% of patients, consistent with findings from previous studies [19, 20]. Biliary tract disease was the most common identifiable source (35%), underscoring the importance of hepatobiliary imaging, preferably with magnetic resonance cholangiopancreatography (MRCP). A German study employing a systematic diagnostic approach, including MRCP and colonoscopy, identified a source in 55% of cases [21]. In our study, a portal vein source was observed in 20% of patients, most commonly associated with diverticulitis, in accordance with the literature [1, 22, 23]. However, in 31% of cases, the source of infection remained unidentified, even after extensive investigations.

The clinical presentation of PLA was variable, with fever and abdominal pain present in approximately half of the patients, whereas jaundice was rare. Importantly, 12% were admitted without classical symptoms, and some of these cases were diagnosed incidentally during scheduled abdominal surgery. This highlights the need for heightened clinical awareness, especially in patients presenting with non-specific complaints or risk factors such as liver tumours or metastases. The median symptom duration before admission was five days, consistent with a previous report [24]. However, the wide range (0–180 days) indicates that some patients experienced prolonged or vague illness, complicating timely diagnosis, suggesting that diagnostic delay remains a concern.

Abnormal laboratory findings were comparable to prior reports, with elevated CRP, leucocytosis, elevated alkaline phosphatase, and hypoalbuminemia being the most prevalent [22, 24, 25]. Imaging with CT confirmed PLA in 96% of patients, with supplementary ultrasound performed in 85% of cases, often post-CT for drainage guidance. MRI and PET-CT were used selectively. Most abscesses were solitary (60%) and located in the right hepatic lobe, with a mean diameter of 5.8 cm, consistent with previous observations [16, 26].

A microbiological diagnosis was achieved by culture in 171 patients (69%). In selected cases, 16S/18S rRNA sequencing provided additional diagnostic value, identifying pathogens in six patients with culture-negative results. As in previous studies, abscess aspirates had a high diagnostic yield [22, 23, 25]. Molecular methods such as 16S rRNA sequencing may further enhance pathogen detection, particularly in patients who have received prior antimicrobial therapy, where conventional cultures are more likely to fail [9, 27]. *E. coli*, the *S. anginosus* group, and *Enterococcus* species were the most frequently isolated pathogens, consistent with previous European findings [9, 16, 27]. Similar patterns were reported in a Norwegian study that utilised both culture and 16S rRNA sequencing [27]. This is likely reflecting the high proportion of PLA cases with biliary origin [28]. *K. pneumoniae* was less common in our cohort; its prevalence varies geographically and is notably higher in populations of Asian descent, where it is often associated with hypervirulent strains [6, 7, 28–30]. *K. pneumoniae* remains clinically important due to its potential for severe disease. Hypervirulent strains such as ST23 (serotype K1) have been reported in Europe, including a Danish case without travel or typical risk factors [31], underlining the need to remain alert for such cases. Polymicrobial infections were frequent, occurring in 33% of positive blood cultures and 54% of positive abscess cultures. Polymicrobial growth in abscess cultures was significantly associated with increased mortality compared to culture-negative abscesses. This finding aligns with a recent Swedish population-based study, which identified polymicrobial infection (OR 3.8), malignancy (OR 3.7), liver failure (OR 6.3), and female sex (OR 2.0) as independent risk factors for 90-day mortality in PLA [32].

Source control with abscess drainage is a critical component of PLA management, and although the optimal duration of antimicrobial therapy is not clearly established, treatment typically ranges from 2 to 6 weeks [1, 3, 16, 33]. In our cohort, piperacillin/tazobactam was the most commonly used agent, followed by a second-generation cephalosporin in combination with metronidazole. However, treatment strategies were also highly variable, reflecting differences in abscess characteristics, microbiology, and clinical course. Due to this heterogeneity, a formal comparative effectiveness analysis of treatment approaches was not performed. Percutaneous intervention was performed in three-quarters of patients, primarily catheter drainage (87%) and less frequently needle aspiration (13%). A recent study by Zhang et al. [34] reported that percutaneous needle aspiration was associated with higher success rates, lower costs, shorter hospital stays, and less procedure-related pain compared with drainage, although both techniques were effective, with primary cure rates above 90%. In our study, non-drainage was not found to be significantly associated with increased mortality. Similarly, a Korean study found that neither late drainage (≥ 48 h after diagnosis due to poor liquefaction) nor non-drainage was associated with higher 90-day recurrence or mortality compared to early drainage [35]. Notably, patients who underwent delayed or no drainage often had smaller abscesses and culture-negative findings. Although no general consensus exists, small abscesses, particularly those with a diameter of less than 3 cm, may be managed successfully with antibiotics alone [36, 37].

In our cohort, the 30-day all-cause mortality rate was 5%, reflecting a substantial improvement compared to earlier national data. Jepsen et al. reported 30-day mortality rates of 15% for men and 23% for women during the period 1977–2002 [2], whereas the 365-day all-cause mortality reached 22%, with no significant difference between sexes. Patients with an infected tumour or metastasis had a markedly higher risk of death, and as discussed previously, polymicrobial growth in abscess cultures was also independently associated with increased mortality. In contrast, drainage, infected liver cysts, multifocal abscesses, and blood culture results were not associated with increased mortality.

A key strength of this study is the comprehensive case identification strategy, which combined registry-based diagnosis codes (ICD-10: K75.0) with microbiological data to enhance case ascertainment. This approach enabled inclusion of an additional 24 patients, nearly 10% of the final cohort, who met predefined diagnostic criteria. The study also draws on a recent validation of the Danish ICD-10 diagnosis code for PLA, which demonstrated a PPV of 77% [11]. The PPV varied by hospital department, with the highest accuracy in medical departments and the lowest in surgical departments. However, relying solely on the discharge diagnosis may reduce sensitivity and risk missing cases. In addition to the robust case identification, the study captures a broad spectrum of clinical, microbiological, and treatment-related information over a 12-year period. However, the retrospective design may have introduced information bias due to incomplete documentation. Additionally, some microbiological data were missing. Blood cultures were not collected in all patients, and abscess cultures were sometimes not obtained, often due to small abscess size or because drainage was not clinically indicated. Furthermore, some cultures were collected after the initiation of antimicrobial therapy, which may have reduced microbiological yield. These factors likely lowered the overall positivity rate and may have led to an underestimation of the proportion of polymicrobial infections. As the study was conducted in a single Danish region, the generalisability of findings to other settings may be limited. Furthermore, although 249 patients were included, PLA remains a relatively rare condition. As such, the study may have limited statistical power for certain analyses, particularly those assessing predictors of mortality, which is reflected in the wide CIs observed for some risk estimates. In addition, even though we adjusted for age, sex, diabetes and cardiovascular comorbidities, other differences between patients, such as underlying health conditions or how quickly and how they were treated, may have affected the results. As such, we cannot exclude residual confounding from unmeasured variables. However, the limited sample size of our study precluded further adjustment of the multivariate models. This underscores the need for larger, preferably multicentre studies to enhance the precision and generalisability of future findings. Nevertheless, the present study offers valuable insights into the clinical management of PLA and may assist clinicians in improving diagnosis and treatment in comparable hospital settings.

## Conclusion

This population-based study describes the epidemiology, clinical features, microbiology, and outcomes of PLA in the North Denmark Region over a 12-year period. The mean annual incidence was 3.1 per 100,000 person-years, with an increasing trend over time. Biliary tract disease was the most common source, though nearly one-third of cases were cryptogenic. While 30-day mortality was low, one-year mortality approached 22%, with underlying malignancy and polymicrobial infections independently associated with worse outcomes. These findings highlight the need for timely diagnosis, microbiological workup, and tailored treatment to improve prognosis in PLA.

## Supplementary Information

Below is the link to the electronic supplementary material.

Supplementary Material 1(DOCX 241 KB)

## Data Availability

The data that support the findings of this study are available from the corresponding author upon reasonable request.
