# Supplementary material for: Pyogenic liver abscess in the North Denmark Region - a population-based cohort study (2010–2022)
Source: Eur J Clin Microbiol Infect Dis. 2025 Oct 17;45(1):287–96. doi: 10.1007/s10096-025-05307-1 (PMC12872774; doi:10.1007/s10096-025-05307-1)
Supplement: Supplementary file 1 — Supplementary Material 1(DOCX 241 KB) [file 10096_2025_5307_MOESM1_ESM.docx]

**Supplementary material**

**Supplementary Figure 1.** Flowchart illustrating the identification and selection of patients with pyogenic liver abscess in the North Denmark Region from 2010 to 2022. Patients were identified through both ICD-10 discharge codes and the regional microbiology database.

**
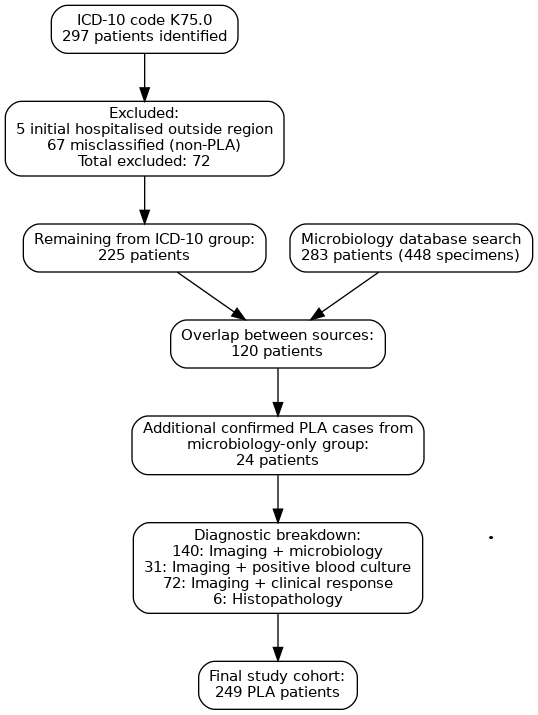
**

**Supplementary Figure 2.** Age distribution among pyogenic liver abscess patients

**Supplementary Figure 3.** Kaplan–Meier survival curves for patients with PLA stratified by abscess culture results. Survival over 365 days following hospital admission is shown for patients with negative abscess cultures (blue line), monomicrobial cultures (red line), and polymicrobial cultures (green line). The number of patients at risk at each time point is indicated below the x-axis.

**
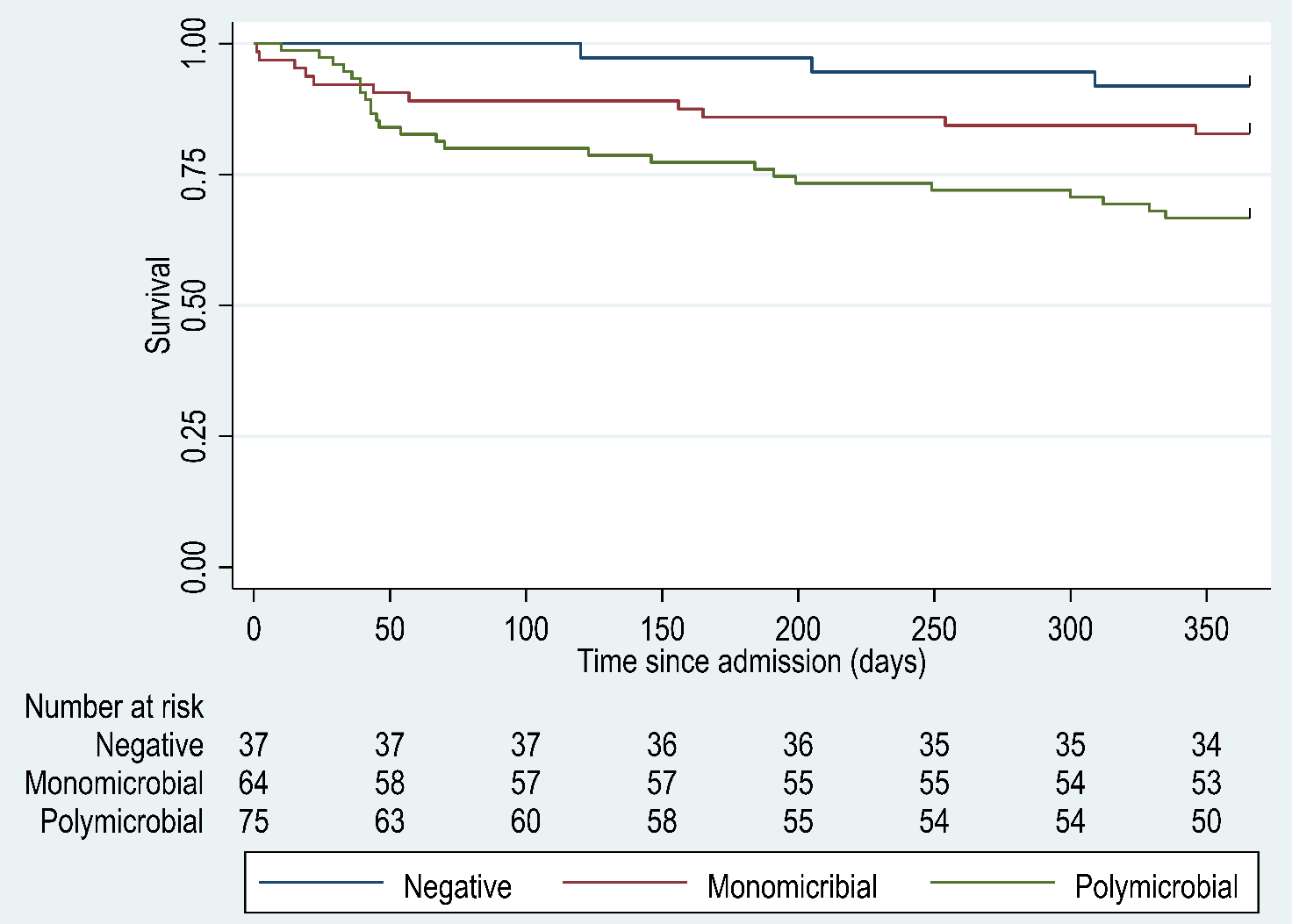
**

**Supplementary Table 1**. Biochemical parameters in patients (n=249) with pyogenic liver abscess.

| Biochemical parameters | **Reference interval** | **Median [IQR]** | **No. of cases outside range (%)** |
| --- | --- | --- | --- |
| White cell count | 3.5 – 10.0 x10^9^ /l | 14.5 (10.7-19.4) | 193 (78) |
| Haemoglobin g/L | Men: 8.3-10.5 mmol/l  Women: 7.3-9.5 mmol/l | 7.3 (6.1-8.0)  7.0 (6.3-7.8) | 107 (78)  63 (55) |
| C-reactive protein | <8 mg/l | 185 (107-270) | 242 (97) |
| Alanine transaminase | 10-70 U/l | 49 (24-86) | 88 (35) |
| Lactate dehydrogenase | 105-205 U/l | 198 (152-256) | 110 (44) |
| Alkaline phosphatase | 35-105 U/l | 174 (114-315) | 194 (78) |
| Bilirubin | 5-25 µmol/l | 14 (8-24) | 65 (26) |
| Albumin | 34-45 g/l | 28 (23-31) | 208 (84) |

**Supplementary Table 2**. Microorganisms found in liver abscess and blood cultures.

| **Bacteria** | **Blood culture (n)** | | **Abscess material (n)** | | **Microbiome sequencing (n)** | |
| --- | --- | --- | --- | --- | --- | --- |
|  | **Mono-microbial** | **Poly-microbial** | **Mono-**  **microbial** | **Poly-**  **microbial** | **Mono-microbial** | **Poly-microbial** |
| **Gram-positive** | | | | | | |
| *Streptococcus anginosus group* | 16 | 2 | 15 | 15 | 1 | 3 |
| Other non-haemolytic *Streptococci* | 2 | 2 | 4 | 3 | 0 | 0 |
| *Streptococcus agalactiae* | 0 | 0 | 0 | 1 | 0 | 0 |
| *Enterococcus faecium* | 1 | 13 | 0 | 15 | 0 | 1 |
| *Enterococcus faecalis* | 1 | 5 | 0 | 11 | 0 | 0 |
| *Enterococcus species* | 1 | 5 | 3 | 12 | 0 | 0 |
| *Staphylococcus aureus* | 2 | 0 | 2 | 0 | 1 | 0 |
| *Staphylococcus epidermidis* | 0 | 0 | 1 | 2 | 1 | 0 |
| *Staphylococcus lugdunensis* | 0 | 0 | 0 | 0 | 1 | 0 |
| *Granulicatella adiascens* | 0 | 0 | 0 | 2 | 0 | 0 |
| *Lactococcus lactis* | 1 | 0 | 0 | 0 | 0 | 0 |
| *Lactobacillus species* | 0 | 2 | 0 | 3 | 0 | 0 |
| *Cutibacterium acnes* | 0 | 1 | 0 | 2 | 0 | 0 |
| *Actinomyces species* | 0 | 0 | 0 | 2 | 0 | 2 |
| Gram-positive cocci  (not specified) | 0 | 0 | 1 | 7 | 0 | 0 |
| Gram-positive rods  (not specified) | 0 | 0 | 1 | 2 | 0 | 0 |
| **Gram-negative** | | | | | | |
| *Escherichia coli* | 16 | 16 | 13 | 32 | 0 | 2 |
| *Escherichia coli* (ESBL^a^) | 1 | 0 | 0 | 3 | - | - |
| *Klebsiella pneumoniae* | 9 | 4 | 9 | 10 | 1 | 0 |
| *Klebsiella pneumoniae* (CPE^b^) | 0 | 1 | 0 | 1 | - | - |
| *Klebsiella oxytoca* | 0 | 3 | 3 | 5 | 0 | 1 |
| *Klebsiella species* | 1 | 2 | 0 | 1 | 0 | 0 |
| *Enterobacter cloacae* | 2 | 3 | 1 | 9 | 0 | 0 |
| *Proteus vulgaris* | 1 | 1 | 0 | 5 | 0 | 0 |
| *Proteus species* | 0 | 1 | 0 | 2 | 0 | 0 |
| *Citrobacter freundii* | 0 | 3 | 0 | 2 | 0 | 1 |
| *Citrobacter freundii* (CPE^b^) | 0 | 1 | 0 | 1 | - | - |
| *Citrobacter* *species* | 0 | 1 | 0 | 0 | 0 | 0 |
| *Morganella morganii* | 0 | 1 | 0 | 1 | 0 | 0 |
| *Hafnia alvei* | 1 | 1 | 2 | 0 | 0 | 0 |
| *Serratia species* | 0 | 1 | 1 | 0 | 0 | 0 |
| *Yersinia enterocolitica* | 1 | 0 | 0 | 0 | 1 | 0 |
| *Pseudomona aeruginosa* | 0 | 3 | 0 | 2 | 0 | 0 |
| *Haemophilus parainfluenzae* | 0 | 0 | 1 | 0 | 0 | 1 |
| *Aeromonas species* | 0 | 1 | 0 | 1 | 0 | 0 |
| *Acinetobacter species* | 1 | 0 | 0 | 0 | 0 | 0 |
| **Obligate anaerobe** | | | | | | |
| *Clostridium perfringens* | 0 | 7 | 0 | 5 | 0 | 2 |
| *Clostridium species* | 1 | 1 | 1 | 0 | 0 | 0 |
| *Parvimonas species* | 1 | 0 | 0 | 1 | 0 | 0 |
| Anaerobic Gram-positive rods (not specified) | 0 | 0 | 1 | 2 | 0 | 0 |
| *Finegoldia magna* | 0 | 0 | 0 | 0 | 0 | 1 |
| *Bacteroides fragilis* | 6 | 3 | 0 | 5 | 0 | 2 |
| *Bacteroides species* | 0 | 1 | 0 | 2 | 0 | 1 |
| *Fusobacterium necrophorum* | 2 | 1 | 1 | 1 | 0 | 5 |
| *Fusobacterium nucleatum* | 2 | 0 | 2 | 0 | 0 | 3 |
| *Prevotella species* | 0 | 0 | 0 | 3 | 0 | 1 |
| *Fretibacterium fastidosum* | 0 | 0 | 0 | 0 | 0 | 1 |
| *Dialister pneumosintes* | 0 | 0 | 0 | 0 | 0 | 1 |
| Anaerobic Gram-negative rods (not specified) | 0 | 0 | 1 | 2 | 0 | 0 |
| Other Anaerobes (not specified) | 0 | 0 | 0 | 11 | 0 | 0 |
| **Fungi** | | | | | | |
| *Candida albicans* | 0 | 1 | 1 | 9 | 0 | 0 |
| *Candida glabrata* | 0 | 2 | 0 | 2 | 0 | 0 |
| *Pichia kudriavzevii* | 0 | 1 | 0 | 2 | 0 | 0 |

^a^Extended-spectrum beta-lactamase

^b^Carbapenemase-producing *Enterobacteriaceae*
